# A phase II trial proposal of total neoadjuvant treatment with primary chemotherapy, stereotactic body radiation therapy, and intraoperative radiation therapy in borderline resectable pancreatic adenocarcinoma

**DOI:** 10.1186/s12885-021-07877-7

**Published:** 2021-02-16

**Authors:** Salvatore Paiella, Giuseppe Malleo, Nicola Simoni, Renato Micera, Stefania Guariglia, Carlo Cavedon, Giovanni Marchegiani, Alessandro Esposito, Luca Landoni, Luca Casetti, Massimiliano Tuveri, Michele Milella, Erica Secchettin, Gessica Manzini, Chiara Bovo, Matteo De Pastena, Martina Fontana, Roberto Salvia, Renzo Mazzarotto, Claudio Bassi

**Affiliations:** 1 Unit of General and Pancreatic Surgery, University of Verona Hospital Trust, Policlinico Rossi, Piazzale L.A. Scuro 10, 37134 Verona, Italy; 2 Unit of Radiation Oncology, University of Verona Hospital Trust, Verona, Italy; 3 Unit of Medical Physics, University of Verona Hospital Trust, Verona, Italy; 4 Unit of Medical Oncology, University of Verona Hospital Trust, Verona, Italy; 5 University of Verona Hospital Trust Management Unit, Verona, Italy

**Keywords:** Intraoperative radiotherapy (IORT), Pancreatic cancer, Neoadjuvant therapy, Locally advanced pancreatic cancer, Stereotactic body radiation therapy (SBRT)

## Abstract

**Background:**

The current management guidelines recommend that patients with borderline resectable pancreatic adenocarcinoma (BRPC) should initially receive neoadjuvant chemotherapy. The addition of advanced radiation therapy modalities, including stereotactic body radiation therapy (SBRT) and intraoperative radiation therapy (IORT), could result in a more effective neoadjuvant strategy, with higher rates of margin-free resections and improved survival outcomes.

**Methods/design:**

In this single-center, single-arm, intention-to-treat, phase II trial newly diagnosed BRPC will receive a “total neoadjuvant” therapy with FOLFIRINOX (5-fluorouracil, irinotecan and oxaliplatin) and hypofractionated SBRT (5 fractions, total dose of 30 Gy with simultaneous integrated boost of 50 Gy on tumor-vessel interface). Following surgical exploration or resection, IORT will be also delivered (10 Gy). The primary endpoint is 3-year survival. Secondary endpoints include completion of neoadjuvant treatment, resection rate, acute and late toxicities, and progression-free survival. In the subset of patients undergoing resection, per-protocol analysis of disease-free and disease-specific survival will be performed. The estimated sample size is 100 patients over a 36-month period. The trial is currently recruiting.

**Trial registration:**

NCT04090463 at clinicaltrials.gov.

## Background

The term “borderline resectable” pancreatic cancer (BRPC) indicates a disease with major venous involvement (solid tumor contact greater than 180 degrees with superior mesenteric vein/portal vein, or contour irregularity of the vein, or thrombosis) and/or arterial abutment (solid tumor contact of less than 180 degrees with superior mesenteric artery and/or celiac trunk) upon thin-slice cross-sectional imaging [1]. The National Comprehensive Cancer Network® guidelines recommend that borderline resectable patients receive primary chemotherapy or chemoradiation, because upfront resection would likely be compromised by positive surgical margins [1]. Although there is no robust evidence to recommend specific chemotherapy regimens off-experimental protocols, the most commonly used regimens include FOLFIRINOX (5-fluorouracil, irinotecan and oxaliplatin) and gemcitabine plus nanoalbumin-bound (nab)-paclitaxel, that have been recently empirically translated from randomized trials of metastatic disease to earlier stages, given the higher response rate relative to gemcitabine alone [2, 3]. In a large observational study of primary chemotherapy for newly diagnosed patients with localized disease, the resection rate in the subgroup of BRPC approached 25%, with the highest rate being reached in patients < 75 years who received FOLFIRINOX (51%). Following resection, the median overall survival was 35.4 months. Cohort-based independent prognostic factors were completion of at least 6 months of chemotherapy, complementary radiation therapy, and resection [4]. This constituted the backbone reference for integrating chemotherapy and radiation therapy into a “total neoadjuvant” approach.

Although in the recent PREOPANC-1 trial preoperative chemoradiotherapy did not show a significant overall survival benefit relative to a surgery-first approach, the outcomes of the secondary end points and predefined subgroup analyses suggest an advantage of the neoadjuvant approach [5]. Remarkably, patients in the experimental arm were treated with an outdated chemotherapy regimen (gemcitabine) and moderately fractionated external-beam radiation (36 Gy administered in 15 fractions). In another single-arm, phase 2 clinical trial conducted at the Massachusetts General Hospital from 2012 to 2016, 48 patients with BRPC received FOLFIRINOX for 8 cycles. Upon restaging, patients with resolution of vascular involvement received short course chemoradiotherapy (5 Gy × 5 with protons) with capecitabine. Patients with persistent vascular involvement received long course chemoradiotherapy with fluorouracil or capecitabine. A margin-negative resection (primary endpoint) was achieved in 65% (intention-to-treat) and 97% of patients (per-protocol). The median progression-free survival (PFS) and OS among all eligible patients were 14.7 and 37.7 months. In patients who underwent resection, the median PFS was 48.6 months, and the median OS was not reached at a median follow-up of 18 months [6]. These latter results confirmed that a total neoadjuvant approach with multiagent chemotherapy and individualized radiation resulted in a high rate of R0 resections and prolonged median PFS and OS.

Advanced radiation therapy techniques, including Stereotactic Body Radiation Therapy (SBRT), have emerged as an attractive solution for BRPC, enabling the delivery of large doses of radiations in fewer fractions relative to the conventional, long-course radiation therapy. In this regard, it has been suggested that preoperative SBRT following up to 6 months of primary chemotherapy might further improve the likelihood of a margin-negative resection and OS over chemotherapy and conventional chemoradiation [7]. At present, the optimal SBRT schedule for BRPC has yet to be determined, with only a few published studies reporting remarkable differences in dose and fractionation [8–11]. Mellon et al. treated 110 BRPC patients with SBRT delivered in 5 consecutive daily fractions with a median total dose of 30 Gy to the tumor and a 40 Gy dose painted to the tumor-vessel interface (TVI) [8]. The R0 resection rate was 96%, and the median OS for resected patients 34.2 months. A recent phase I trial investigated a total neoadjuvant approach with FOLFIRINOX followed by dose escalated SBRT [11]. The maximum tolerated dose of SBRT was 36 Gy in 3 fractions to the tumor, with a boost to the posterior margin (defined as the area between the posterior 1 cm of the tumor and mesenteric vessel/retroperitoneal soft tissue) up to 45 Gy in 3 fractions. The R0 resection rate was 66.6%, the median OS for resected patients was not reached, and no grade ≥ 3 adverse events were reported. In a recent observational study from the authors’ Institution, 88.9% of BRPC patients received surgical resection following multiagent chemotherapy and SBRT with simultaneous integrated boost (SIB) and simultaneous integrated protection (SIP) [12]. The prescribed dose was 50/30/25 Gy in 5 consecutive daily fractions to TVI (SIB)/planning target volume (PTV) tumor/overlap area between PTV and organs at risk (SIP), respectively. No acute or late grade ≥ 3 SBRT-related adverse events were observed. However, about 40% of patients experienced an R1 resection. In more than 70% of R1 patients, microscopic residual occurred at the posterior margin/retroperitoneal lamina, and the performed dosimetric evaluation showed a predominant incidence of in-field recurrence, with a recurrence median dose of 40.42 Gy. In this respect, current recommendations support the application of intraoperative radiation therapy (IORT) as a boost strategy (integrated for a dose-escalation multimodality approach) to maximize local control and impact upon survival [13].

IORT is the application of a single fraction of radiation therapy to the target volume at the time of surgery while sparing the surrounding organs. Data regarding IORT in PC have been evaluated in retrospective single- or multi-institutional series, showing 40–80% reduction of loco-regional recurrence as compared to the standard treatment [14]. When applied to PC, IORT was associated to consistent benefits in terms of overall survival, either in the surgery plus IORT group, and in the locally advanced PC group, where IORT was the only local treatment [15–18].

In the light of the most recent results of multiagent chemotherapy regimens and radiation therapy techniques, we herein propose a phase II trial of total neoadjuvant treatment with FOLFIRINOX, hypofractionated SBRT with SIB/SIP, and IORT (following the resection phase, or as an in situ treatment) in newly diagnosed BRPC.

## Methods/design

The study reporting adheres to the SPIRIT Guidelines [19]. This single-arm phase II trial will be conducted at the University of Verona Hospital Trust and will enroll newly diagnosed patients with BRPC. The trial has been registered at www.clinicaltrials.gov (NCT04090463) and has been approved by the local Ethics Committee (#2301CESC). The expected total study duration is 72 months (36 months for enrollment and 36 months for follow-up).

### Endpoints

The primary endpoint is the 3-year disease-specific survival (intention-to-treat). Secondary endpoints include:

- Completion of chemotherapy,
- Completion of SBRT,
- Acute and late toxicities,
- Resection rate,
- Perioperative morbidity and mortality,
- Rate of margin-free surgery,
- Progression-free survival (local/distant progression), intention-to-treat,
- Recurrence-free survival in the subset of patients undergoing resection (per-protocol),
- 3-year disease-specific survival in the subset of patients undergoing resection (per-protocol).

### Study population

#### Inclusion criteria

- Biopsy-proven, previously untreated BRPC, defined according to the NCCN guidelines v3.2019 [1],
- Age 18–75 years,
- Eastern Cooperative Oncology Group (ECOG) performance status 0 or 1,
- Adequate bone marrow function (absolute neutrophil count ≥1500 per cubic millimeter; platelet count ≥100.000 per cubic millimeter; hemoglobin level ≥ 10 g per deciliter), liver function (serum total bilirubin level ≤ 1.5 times the upper limit of the normal range), and renal function (creatinine clearance ≥50 ml per minute),
- Ability to understand the characteristics of the clinical trial,
- Written informed consent.

#### Exclusion criteria

- Ampullary, biliary, or duodenal adenocarcinoma; pancreatic adenocarcinoma in the background of an intraductal papillary mucinous neoplasia (IPMN), other uncommon pancreatic adenocarcinomas (acinar-cell, squamous, giant-cell osteoclastic-like),
- Primary tumor > 60 mm,
- Invasive cancer in the last 5 years requiring radiation therapy to the upper abdomen or chemotherapy,
- Symptomatic heart failure or coronary artery disease,
- Pregnant or lactating women,
- Impaired mental state or language problems.

### Procedures

#### Diagnostic assessment and treatment allocation

PC is diagnosed using thin-slice cross-sectional imaging (contrast-enhanced tri-phasic computed tomography of the thorax and abdomen) or gadolinium-enhanced magnetic resonance. 18-FDG positron-emission tomography is performed to rule out suspected distant metastases (indeterminate liver/pulmonary lesions on cross-sectional imaging). Tissue diagnosis is performed via endoscopic ultrasound or transabdominal ultrasound guided fine-needle aspiration. Baseline serum carcinoembryonic antigen (Ca) 19.9 is measured at the time of diagnosis, per standard practice. In jaundiced patients, endoscopic retrograde cholangiopancreatography (ERCP) is performed, and self-expandable fully covered metal biliary stent is placed. Ca 19.9 assessment is repeated following biliary drainage (ideally when bilirubin drops to < 3 mg/dL). All newly diagnosed patients with BRPC will be collegially evaluated in a multidisciplinary meeting (The Verona Pancreas Institute Oncologic meeting) and screened for inclusion by dedicated Oncologists, Radiation Oncologists, and Surgeons.

#### Chemotherapy

Following informed consent, FOLFIRINOX will be administered either in Verona or in local hospital operating within a regional oncologic network on a 14-day cycle for up to 12 cycles (minimum 8). Fluorouracil will be administered as a 400-mg/m2 bolus on day 1, then as a 2400 mg/m2 continuous infusion for 46 h. Leucovorin calcium, 400 mg/m2; oxaliplatin, 85 mg/m2; and irinotecan hydrochloride, 180 mg/m2, will be administered on day 1. Protocol-specified treatment modifications will be undertaken when significant toxic effects occur according to the Common Terminology Criteria for Adverse Events, version 4.0. Treatment discontinuation for severe toxicity or performance status deterioration/disease progression will be recorded. Patients discontinuing chemotherapy will not be considered for SBRT and will be included in the intention-to-treat analysis.

#### Restaging and indications for SBRT and surgical exploration

Patients completing chemotherapy will be re-staged with thin-slice contrast-enhanced tri-phasic computed tomography of the thorax and abdomen and re-evaluated at the Verona Pancreas Institute Oncologic meeting. Gadolinium-enhanced magnetic resonance will be considered in case of undetermined liver lesions (e.g. < 1 cm). Protocol prosecution will be indicated if surgical resection will be considered feasible. The minimum requirements for surgical eligibility will be partial response or stable disease according to the response evaluation criteria in solid tumors (RECIST) v1.1 [20] and a performance status of 0–1 (ECOG). Serum Ca 19.9 levels will be also considered in the decision process. An increase of Ca 19.9 values relative to the baseline will be considered a possible sign of progressive disease. Post-treatment values > 1000 U/L will be considered suspicious of micro-metastatic disease. In the instance of stable disease per RECIST criteria and increasing Ca 19.9 diagnostic laparoscopy will be considered.

SBRT will be considered only in surgical candidates, provided that:

- There are no suspicious lymph nodes outside the planning target volume (PTV),
- There is no duodenal/gastric infiltration.

Patients not eligible for surgical exploration will not be considered for protocol prosecution, will be re-evaluated for possible second-line chemotherapy or maintenance chemoradiation, and will be included in the intention-to-treat analysis.

#### SBRT

SBRT will be planned within 4 weeks from chemotherapy completion. Fiducial markers will be placed via EUS prior to CT-simulation. SBRT will be delivered only at the Verona Pancreas Institute as follows: patients are immobilized in supine position with arms over the head, using a custom-made Vac-Lok™ cushion. Abdominal compressor or inspiration breath hold (IBH) technique are used to manage breathing induced tumor motion. To give respiratory feedback to the patient a Real-Time Position Management® system (Varian Medical Systems, Palo Alto, CA) will be used. The gross tumor volume (GTV) is delineated as the radiographically evident gross disease on a multi-phases contrast-enhanced simulation CT. An internal target volume (ITV) will be defined as the envelope of the GTVs from each CT-phase. An ITV to Planning Target Volume (PTV) margin of 5 mm is applied. For organs at risk such as duodenum, stomach and bowel, a 3 mm expansion planning organ at risk volume (PRV_oar_) is defined. In case of proximity of the target volume to organs at risk, a simultaneous protection volume (PTV_sip_) will be generated as the intersection of the PTV and the PRV_oar_. A simultaneous integrated boost (SIB) to the tumor vessel interface (TVI) is planned. SBRT plans will be delivered using RapidArc® technique (Varian Medical Systems, Palo Alto, CA, USA) by a True Beam® linear accelerator (Varian Medical Systems, Palo Alto, CA, USA) with a 6-MV flattening filter-free (FFF) photon beam energy to minimize treatment duration. The standard schedule involves delivery of 30 Gy in 5 consecutive daily fractions to the PTV, with a boost on the TVI up to 50 Gy (accepted range 40–50 Gy, corresponding to a biologically effective dose (BED_10_) of 72–100 Gy). The prescribed dose will be reduced to 25 Gy (5 Gy/fraction) on the PTV_sip_, in order to avoid toxicities. Normal tissue constraints are as follows: for duodenum, small bowel and stomach Dmax < 35 Gy, V30 Gy < 5 cc, Dmean < 20 Gy; for spinal cord Dmax < 20 Gy; for kidneys Dmean < 10 Gy and D200cc < 17,5 Gy; for liver D700cc < 21 Gy. On-line image guidance (IGRT) will be performed by means of cone beam computed tomography (CBCT) before every single treatment session.

Patients completing SBRT will be re-staged with thin-slice contrast-enhanced tri-phasic computed tomography to rule out distant progression and will be further re-evaluated at the Pancreas Institute Oncological multidisciplinary meeting.

#### Intraoperative determinants of resectability

All surgeries will be carried out at the Verona Pancreas Institute. Determinants of intraoperative resectability will be:

- Absence of distant metastases,
- Absence of portal vein cavernoma (unreconstructible venous anatomy),
- No need for superior mesenteric artery resection.
- Acceptable arterial resections include common hepatic artery resection (performed only when a short segment – up to 2 cm – is involved), and Appleby or modified Appleby procedure (performed for solid tumor contact with celiac trunk, without aortic involvement) [21].

#### IORT

IORT will be delivered in a dedicated hybrid operating room with a portable linear accelerator (Mobetron®, IntraOp Medical Corporation, Sunnyvale, CA, USA) provided with a set of collimator (collimator diameter ranging from 3 to 10 cm (step 0.5 cm) each one with bevel angle of 0°, 15° or 30°). Following the resection phase, the radiation oncologist and the surgeon will decide the collimator dimension and the angle of bevel, according to post-surgical anatomy and tumor bed characteristics. For unresectable tumors, collimator dimension and bevel angle will be defined after surgical exploration according to tumor dimensions. Collimator dimension will be selected to encompass with a 1-cm margin the resection bed or in situ tumor. Guidelines for radiation dose are as follows: resection bed 10–15 Gy; unresectable tumor 13–20 Gy. In case of unresectable head/neck tumors a gastrojejunostomy will be performed (to avoid a possible gastric outlet obstruction due to bowel edema). A cholecystectomy may be performed if indicated.

### Postoperative management

Patients will be managed according to our standard practice. Procedure-specific complications, including pancreatic fistula, post-pancreatectomy hemorrhage, delayed gastric emptying, and chyle leak will be defined according to the International Study Group of Pancreatic Surgery recommendations [22–24]. Infectious, pulmonary, cardiac, neurologic, and renal complications will be also recorded. Postoperative complications will be graded according to the Clavien-Dindo classification [25]. Hospital readmission and 90-day postoperative mortality will be recorded.

### Follow-up

#### Efficacy assessment

Patients will be followed for 36 months postoperatively or from the time of early progression/surgical ineligibility. Follow-up visits will take place every 3 months after surgery for the first 12 months, then on a 6-month basis up to study completion/death (Table 1). In patients with disease progression during the neoadjuvant phase and in those who were not eligible for surgical exploration, follow-up visits will be scheduled on a 6-month basis. The surveillance protocol includes a thin-slice contrast-enhanced, tri-phasic computed tomography, Ca 19.9 measurement and a thorough clinical examination. Complementary gadolinium-enhanced magnetic resonance and/or 18-FDG positron-emission tomography will be performed in selected cases, following multidisciplinary consultation. Disease recurrence (either local or distant) is defined as the presence of biopsy-proven tumor or was assumed based on the above-mentioned cross-sectional imaging studies, in conjunction with clinical picture and/or serum Ca 19.9 levels.

**Table 1:** Flow-chart of study procedures

	Pre-study	PC completion	SBRT completion	Surgery + IORT^**a**^	Follow-up							
Assessments												
Informed consent	X				3 mo	6 mo	9 mo	12 mo	18 mo	24 mo	30 mo	36 mo
Physical evaluation	X	X	X	X	X	X	X	X	X	X	X	X
AE assessment		X	X	X	X	X	X	X	X	X	X	X
Ca 19–9, CEA	X	X	X	X	X	X	X	X	X	X	X	X
CT-scan (thorax/abdomen)	X	X	X		X	X		X	X	X	X	X
MRI	Optional	Optional	Optional									

*PC* primary chemotherapy, *IORT* intraoperative radiation therapy

^a^at the time of hospital discharge

### Statistical considerations

#### Sample size calculation

The estimate of accrual was calculated hypothesizing that total neoadjuvant therapy with FOLFIRINOX + SBRT + IORT will improve the 3-year survival rate up to 37% in comparison with cohort-level institutional data (patients < 75 years with BRPC undergoing neoadjuvant FOLFIRINOX, 3-year survival of 23.7%) Accrual time was set at 36 months and follow-up at 36 months. At a power of 0.90, and alpha of 0.5 a sample size of 100 patients was calculated assuming a non-parametric estimate of survival distribution. The SWOG Cancer Research and Biostatistics one-arm trial with time-to-event data sample size calculator was employed [26].

#### Survival analysis

The progression-free survival (for patients who will not ultimately receive pancreatectomy due to unexpected locally unresectable or metastatic disease) and the disease-specific survival (for patients receiving pancreatectomy) will be calculated from both the date of diagnosis and the date of last follow-up or recurrence/death from disease, using the Kaplan-Meier method. Pairwise differences in survival will be assessed using the log-rank test. The analysis of recurrence and survival predictors will be carried out using the proportional hazard Cox regression (backward stepwise elimination technique, Wald method, *P* < .05 for entry, *P* > .10 for removal). Statistical significance will be determined by a 2-sided *P* value less than .05. Data will be using SPSS software (IBM).

### Safety assessment

#### Adverse event reporting

The descriptions and grading scales found in the revised NCI Common Terminology Criteria for Adverse Events (CTCAE) version 4.0 [27] will be utilized for adverse event reporting.

#### Expected adverse event reporting

Investigators must report to the PI and Coordinating Center any adverse event (AE) that occurs after the initial study treatment, during treatment, or post treatment, within 3 years of follow-up on the SAE form. This reporting requirement applies to any medical equivalent to:

- an unexpected grade 2 or 3 event* with a possible, probable or definite attribution
- any grade 4 event, and
- grade 5 (death) regardless of attribution

*Grade 2 and Grade 3 laboratory abnormalities that are considered by the investigator to be.

clinically insignificant and do not require therapy, or adjustment in prior therapy, do not need to.

be reported.

#### Routine adverse event reporting

All adverse events, whether reported by the participant, discovered during questioning, directly observed, or detected by physical examination, laboratory test or other means, must be reported in routine study data submissions to the Overall PI on the toxicity case report forms. Adverse events reported through expedited processes (e.g., reported to the IRB) must also be reported in routine study data submissions.

### Data collection and ethical considerations

The trial will be conducted according to the principles of the Declaration of Helsinki (64th version, October 2013) and in accordance with the local laws and regulations. The local principal investigator is responsible for making sure that local laws and regulations are followed.

All subjects will be recruited at the outpatient clinic by one of the principal investigators. The principle investigator may be replaced by an assigned substitute, who is fully informed and aware of the study requirements and procedures, e.g. the study coordinator or local treating physician. Patients enrolled, will be coded by a numeric code (deidentified), the principal investigator and co-investigators will be the only one with access to it. The source data will be stored digitally and will be kept by the project leader for 25 years after the inclusion of the last patient. The study will be conducted in compliance with following documents: European Directive 91/507 / EEC, Decree 211/03, D.M. December 21, 2007, AIFA Determination March 20, 2008, D.L. 189 of 08/09/2012, European Directive 2011 / C172 / 01 and Decree 2016/679. The evaluation of the study is the responsibility of the Ethics Committee, and its implementation will be possible only after its approval. Any amendment to the Protocol will follow the same approval process.

## Discussion

This phase II trial will primarily investigate the outcomes of a total neoadjuvant approach combining multiagent chemotherapy (FOLFIRINOX), hypofractionated SBRT with SIB/SIP followed by surgical resection and IORT in BRPC. Patients will be screened and enrolled at the time of diagnosis at the University of Verona Hospital Trust, a national referral center for pancreatic cancer. Because most patients referred to our center live a considerable distance away, neoadjuvant FOLFIRINOX will be administered either in Verona or at local institutions operating within a regional oncologic network, according to the patient’s area of residence. Information on the ongoing treatment will be acquired from local centers by dedicated data managers. Patients will be restaged at the coordinating center, indication to surgical exploration will be based on the evaluation of RECIST response, Ca 19.9 response and performance status by a multidisciplinary panel. SBRT, surgeries and IORT will be carried out at the Verona Pancreas Institute. Preliminary institutional experience showed that hypofractionated SBRT with SIB/SIP is feasible and is not associated with toxicity/adverse events that preclude subsequent surgical resection [12]. The addition of IORT could further reduce the recurrence or progression of the disease, ultimately impacting positively on prognosis [28]. Even the safety profile of IORT seems very favorable, with only few cases of > grade 2 acute toxicities having been reported so far in the literature [18]. Finally, in previous experiences patients undergoing pancreatic resection after neoadjuvant therapy exhibited a reduced incidence of postoperative complications, including postoperative pancreatic fistula and post-pancreatectomy hemorrhage [29].

The main strength of this study is the intention-to-treat design, in such a way that cohort-level data on recurrence and survival will be available. Furthermore, secondary endpoints including completion of neoadjuvant treatment, resection rate, toxicity, and progression-free survival can be addressed. In the subset of patients undergoing resection, per-protocol analysis of disease-free and disease-specific survival will be performed. The trial is currently recruiting. Enrollment is expected to be completed within 36 months from the study kick-off.

## Data Availability

The datasets generated and/or analyzed during the current study are not publicly available for Institutional policy, but are available from the corresponding author on reasonable request.

## Abbreviations

PC: pancreatic cancer
IORT: intraoperative radiotherapy
SBRT: stereotactic body radiation therapy
PFS: progression-free survival
GTV: gross tumor volume
ITV: internal target volume
PTV: planning tumor volume
PRV: planning organ at risk volume
TVI: tumor vessel interface
AE: adverse event
